# Delayed diagnosis of spinal osteoblastoma presenting with radicular pain and scoliosis: A case report

**DOI:** 10.1016/j.ijscr.2025.110924

**Published:** 2025-01-22

**Authors:** Faten Limaiem, Mouadh Nefiss, Ramzi Bouzidi

**Affiliations:** a University of Tunis El Manar, Faculty of Medicine of Tunis, 1007, Tunisia; b Pathology Department, Hospital Mongi Slim La Marsa, Tunisia; c Department of Orthopedic Surgery, Hospital Mongi Slim La Marsa, Tunisia

**Keywords:** Osteoblastoma, Lumbar spine, Pathology, Orthopedics, Bone tumor, Case report

## Abstract

**Introduction and importance:**

Osteoblastoma is a rare benign bone tumor, accounting for 1 % of primary bone tumors, often affecting the spine and sacrum. Accurate diagnosis is essential for appropriate treatment and prognosis.

**Case presentation:**

A 19-year-old male presented with two years of persistent nocturnal radicular and low back pain unresponsive to anti-inflammatory medications. Physical examination revealed a left-sided gibbosity and a positive Sonnette sign at lumbar levels L3-L4 and L4-L5 without neurological deficits. MRI and CT scans revealed anomalies in the right facet joint at L3-L4 and a lytic lesion at the L3 inferior articular process, suggestive of osteoblastoma. The patient underwent *en bloc* resection of the right L3 inferior articular process, decompression of the right L3 root, and tumor curettage. A unilateral fixation with pedicle screws was performed to prevent instability. Histological examination confirmed osteoblastoma. The patient's postoperative recovery progressed moderately, and he is actively participating in physical therapy, with continued follow-up planned to monitor for any potential recurrence or complications.

**Clinical discussion:**

Osteoblastoma diagnosis is based on clinical, radiological, and histopathological evaluation. It is important to distinguish osteoblastoma from similar tumors for appropriate management. Surgical intervention, including *en bloc* resection or curettage, is the treatment of choice based on clinical factors and tumor location.

**Conclusions:**

This case highlights the challenges in diagnosing spinal osteoblastoma, especially in young patients with persistent back pain. Early recognition, prompt intervention, and surveillance are critical for optimal outcomes.

## Highlights

• Osteoblastomas are rare benign bone tumors, constituting about 1% of all bone tumors.
• They are classified into conventional and aggressive types based on presentation, imaging, and histology.
• They cause progressive pain, bone destruction, and can lead to neurological and radicular symptoms, especially in the spine.
• Radiological assessment helps characterize the lesion, but diagnosis requires histopathological confirmation.
• Recurrence of osteoblastoma occurs in 15-25% of cases, particularly after curettage.

## 1 Introduction

Osteoblastoma is a rare, benign bone-forming tumor, accounting for approximately 1 % of primary bone tumors and 1–5 % of benign bone tumors. It represents 10 % of osseous spinal neoplasms [1]. The vertebral column is the most common site, with osteoblastomas found in 28–36 % of cases, while long bones are the second most frequent location [2]. These tumors are classified into two types: conventional and aggressive osteoblastomas, based on clinical presentation, imaging features, and histology [2]. Accurate diagnosis, which relies on clinical evaluation, radiology, and histopathological examination, is essential for determining the appropriate treatment and prognosis [3]. Due to their rarity, diagnosing and treating osteoblastomas, particularly in the lumbar spine of young adults, presents unique challenges. Herein, we report a rare case of osteoblastoma in the lumbar spine of a 19-year-old male, aiming to address the diagnostic and therapeutic uncertainties associated with this uncommon tumor. This case report adheres to the SCARE Criteria [4].

## 2 Case presentation

**Fig. 1:**
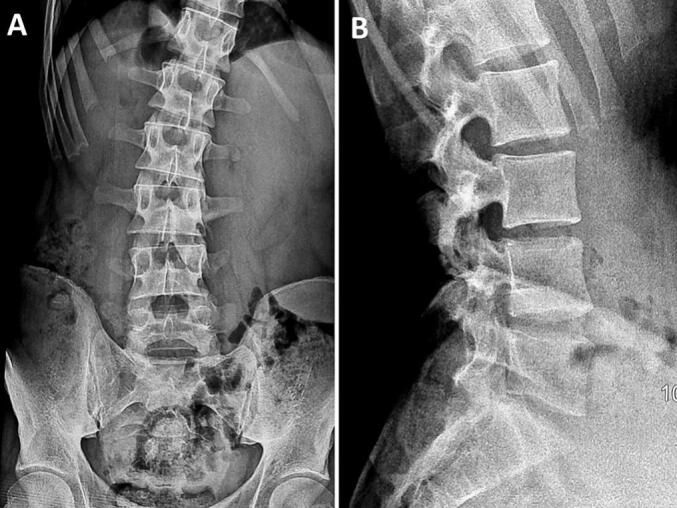
(A, B): Anteroposterior (A) and lateral view (B) radiographs illustrating a distinctive lytic lesion positioned at the right inferior articular process of L3.

**Fig. 2:**
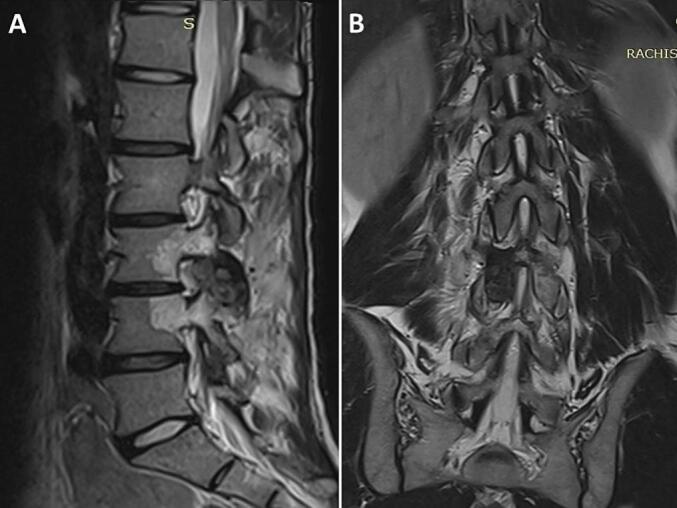
(A, B): The MRI depicted edematous signal abnormalities in the right lateral and posterior arches of the L3 and L4 vertebrae, characterized by hyperintensity on T2 and STIR sequences and hypointensity on T1. Post Gadolinium injection, these signals exhibited homogeneous enhancement. The abnormalities were centered on the ipsilateral posterior zygapophyseal joints, notably enhancing the right L4 zygapophyseal joint space, and extended into the right lumbar psoas and paravertebral muscles, without evident collections.

**Fig. 3:**
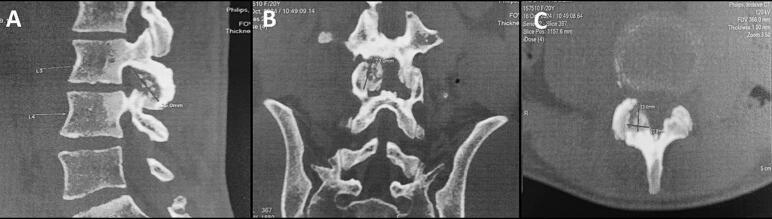
(A, B, C): CT scan showing a well-defined lytic lesion located at the right inferior articular process of L3, extending to the inferior articular surface of L3 and the adjacent superior articular process of L4. The lesion includes a calcified nidus causing cortical disruption, surrounded by osteocondensation with blurred borders.

**Fig. 4:**
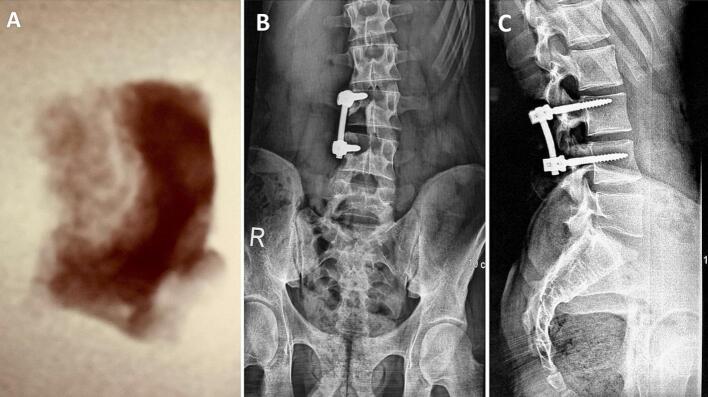
Specimen radiograph (A) and Postoperative X-rays depicting the extent of resection and successful placement of pedicle screws, as seen in the anteroposterior view (B) and lateral view (C).

**Fig. 5:**
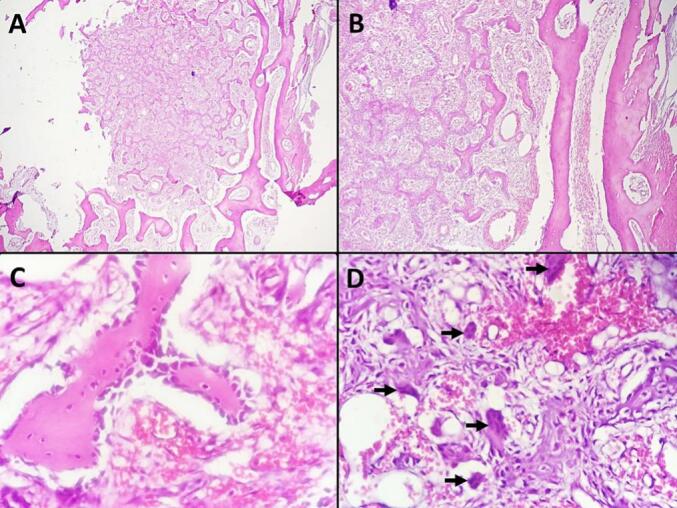
A. Low-power view of Osteoblastoma with a well-defined tumor border. The bony lesion is encircled by a sclerotic rim and consists of inter-anastomosing trabeculae of woven bone within a loose, edematous fibrovascular stroma. (Hematoxylin and eosin stain, magnification ×40). B. Bony lesion composed of inter-anastomosing trabeculae of woven bone, set within loose edematous fibrovascular stroma, with extravasated erythrocytes. **(**Hematoxylin and eosin, magnification ×100**)**. C. High-power view of Osteoblastoma: Osseous trabeculae bordered by a single layer of activated osteoblasts within a loose, edematous fibrovascular stroma. (Hematoxylin and eosin stain, magnification ×400). D. High-power view of Osteoblastoma showing scattered osteoclast-type, multinucleated giant cells (black arrows). (Hematoxylin and eosin stain, magnification ×400).

A 19-year-old man with no significant medical history presented with a two-year history of nocturnal right lumbar paravertebral radicular and low back pain unresponsive to anti-inflammatory medications. The patient denied any history of trauma, deterioration in overall health, or associated neurological symptoms. Physical examination revealed the patient to be in good general condition, with stable gait and no limping observed during ambulation. A left-sided gibbosity was noted, with a positive Sonnette sign at the lumbar spine levels L3-L4 and L4-L5 on the right. Straight leg raising test was negative. No neurologic deficit was noticed. X-rays (Fig. 1), requested six months prior to diagnosis, displayed a lytic image on the right inferior articular process (IAP) of L3 and a left convex scoliotic posture. The patient was provided with symptomatic treatment, and no further investigations were requested. Due to the lack of improvement, the patient sought our consultation, leading to an MRI (Fig. 2) showing an edematous signal abnormality in the right lateral and posterior arches of the L3 and L4 vertebrae. This anomaly exhibited hyperintensity on T2 and STIR sequences and hypointensity on T1, enhancing homogeneously following Gadolinium injection. These signal irregularities were focused on the ipsilateral posterior zygapophyseal joints, with enhancement of the right L4 zygapophyseal joint space. The signal abnormalities extended to the right lumbar psoas and paravertebral muscles without any distinguishable collection within them. A CT scan (Fig. 3) unveiled a well-defined lytic lesion centered on the right inferior articular process of L3, extending to the inferior articular surface of L3 and the adjacent superior articular process of L4. A calcified nidus causing cortical disruption was surrounded by osteocondensation with indistinct borders, confirming the diagnosis of spinal osteoblastoma. Under general anesthesia, the patient underwent *en bloc* resection of the right L3 IAP, with the surgical approach carefully planned to ensure adequate exposure and minimize risks. The procedure began with precise osteotomy at the level of the right lamina and isthmus of L3, following established anatomical landmarks for safe dissection. Intraoperative findings revealed more extensive tumor involvement than anticipated on preoperative imaging, with invasion into three critical areas: the joint space, the proximal part of the superior articular process, and the L3-L4 right foramen. This discovery required adaptation of the surgical plan, demonstrating the importance of having a complete array of surgical equipment available. A meticulous curettage was performed with particular attention to protecting the L3 nerve root, which was found to be in close proximity to the tumor. To address potential instability following the resection, unilateral fixation with pedicle screws at L3-L4 was performed (Fig. 4). The procedure required careful consideration of both planned and unplanned morbidity risks, with particular attention to preventing complications. The surgery concluded with meticulous hemostasis and closure, recognizing that technical problems or complications could lead to persistent postoperative pain. The resection specimen was sent to the pathology laboratory for analysis. Histological examination confirmed the diagnosis of osteoblastoma (Fig. 5).

The patient's postoperative recovery progressed moderately. He was prescribed physical therapy, which he actively participated in, and received ongoing outpatient follow-up care, including a six-week monitoring period. A comprehensive follow-up plan was established to closely monitor for any potential recurrence or late complications.

## 3 Discussion

The World Health Organization classifies osteoblastomas as benign bone-forming tumors that exhibit local aggressiveness, sharing morphological similarities with osteoid osteoma. However, they possess a greater growth potential and typically exceed 2 cm in size [1,5]. The spine, specifically the neural arch (posterior elements), is the most commonly affected site, observed in over a third of cases. While these tumors can spread into the vertebral body, isolated occurrences within the vertebral body itself are exceedingly rare [1,5]. In our case, the CT scan identified a well-demarcated lytic lesion originating from the right inferior articular process of L3, with extension into the inferior articular surface of L3 and the superior articular process of L4. Spinal osteoblastomas predominantly affect the pediatric population, especially children aged 10 to 15 years, with a male-to-female ratio of approximately 2:1 [1,6]. Our patient was a 19-year-old male. The precise etiology of osteoblastoma remains unknown, as do any identifiable predisposing factors. Osteoblastomas typically manifest as solitary lesions and are seldom found to be multicentric [7]. The clinical symptoms of osteoblastomas are nonspecific. A key feature is progressive localized pain that does not respond to anti-inflammatory medications [1,6]. In cases of spinal osteoblastoma, additional symptoms may include radicular pain, scoliosis, and neurological deficits [6,7]. Due to the rarity and nonspecific nature of symptoms associated with spinal osteoblastoma, these lesions are frequently diagnosed in advanced stages rather than early in the disease course [7]. Our patient presented with a two-year history of nocturnal right lumbar radicular and low back pain, unresponsive to anti-inflammatory medications, along with scoliosis.

Osteoblastoma can lead to significant bone destruction, infiltration of soft tissues, and extension into the epidural space, posing challenges for surgical treatment [1,2]. Neurological deficits, such as paraparesis and paraplegia, are present in around a third of patients, while radicular symptoms may affect up to half of patients due to factors like pathological fractures or soft tissue expansion causing pressure [1,2,8]. Radiological assessment characterizes and determines lesion extent, but diagnosis requires histopathological examination [1]. Plain radiographs are initial steps in spinal surgery cases [1,9], yet may overlook small lesions in complex spinal areas due to bone overlap. Whole-Body Bone Scintigraphy is a sensitive diagnostic tool for osteoblastoma [10]. CT scans are crucial for identifying calcification, mineralization, and bone destruction, aiding preoperative planning [1,8], preferred over plain radiography. CT scan typically shows a lytic expansile lesion encased in a sclerotic shell with soft tissue masses [1,8], superior to MRI in revealing calcification or ossification [1,8]. MRI features for spinal osteoblastoma are nonspecific, showing low to isointense signals on T1-weighted and intermediate to high signals on T2-weighted sequences [1,8]. The treatment of osteoblastoma remains debated, with surgery as the primary approach. Extensive surgical resection is essential for optimal outcomes and preventing local recurrence. While total *en bloc* resection is preferred, when possible, it may not be feasible in spinal lesions due to anatomical constraints, tumor size, and potential morbidity [3,6]. Surgical indications for spinal osteoblastoma include persistent pain, tumor growth, neurological deficits, and risk of malignant transformation or bony destruction compromising spinal stability. The choice between *en bloc* resection and curettage depends on the clinical scenario, tumor location, and malignancy suspicion. En bloc resection is favored when viable, as it reduces recurrence risk, while precise intraoperative localization and thorough resection are key to achieving favorable clinical and radiological outcomes [3,6]. In our case, the patient underwent *en bloc* resection of the right L3 inferior articular process, following osteotomy of the ipsilateral lamina and isthmus. This was combined with decompression of the right L3 nerve root and tumor curettage, which had infiltrated the L3-L4 foramen and partially involved the superior articular process of L4. To prevent instability, a unilateral pedicle screw fixation was performed. Radiation therapy for spinal osteoblastomas is controversial, with concerns about its potential to induce sarcomatous transformation and limited effectiveness. It is occasionally used as an adjunct after intralesional curettage of Stage 3 lesions not suitable for *en bloc* resection [1,6]. Our patient did not undergo radiotherapy. The definitive diagnosis relies on pathological examination, considered the gold standard in confirming osteoblastoma [7,11]. Histologically, osteoblastomas are characterized by woven bone trabeculae in a fibrovascular stroma, with or without a sclerotic nidus [11]. Bony maturation includes osteoblast clusters and trabeculae [1,11], with osteoblast lining and osteoclastic giant cells indicating remodeling [11]. The differential diagnoses of osteoblastoma include osteoid osteoma, aneurysmal bone cyst, giant cell tumor of bone, osteoma with osteoblastoma-like features, and osteoblastoma-like osteosarcoma [11]. The prognosis for osteoblastoma is generally favorable, with most patients cured following initial surgical treatment. However, local recurrence is common, particularly after curettage, with rates ranging from 15 % to 25 % [12,13]. Recurrence peaks within the first two years post-treatment. Misdiagnosis of aggressive osteoblastoma or osteoblastoma-like osteosarcoma can lead to delayed and incomplete treatment. Long-term monitoring, including imaging and clinical follow-up for at least two years, is standard practice due to the risk of recurrence [14]. Malignant transformation from osteoblastoma to osteosarcoma, though rare, has been reported but remains controversial [12,14]. In our case, the patient's postoperative recovery proceeded without immediate complications. However, given the potential for recurrence and the long-term outcomes associated with spinal osteoblastoma, the follow-up period should be extended to at least 6 to 12 months. This extended monitoring will provide a more comprehensive evaluation of the surgical outcome and allow for early detection of any recurrence or late-onset complications.

## 4 Conclusion

In summary, this case report of spinal osteoblastoma highlights several unique aspects, including a two-year delay in diagnosis and the development of secondary scoliosis and radicular pain. These findings underscore the challenges in recognizing this rare tumor, particularly in young patients with non-specific symptoms. The prolonged diagnostic delay emphasizes the importance of maintaining a high index of suspicion, particularly in cases of persistent back pain, and highlights the need for thorough clinical evaluation and advanced imaging studies to ensure prompt diagnosis. This case report contributes to the literature by emphasizing the importance of early recognition, prompt intervention, and ongoing surveillance to improve patient outcomes and prevent complications associated with spinal osteoblastoma. Ongoing research is essential to refine diagnostic methods and treatment strategies, ultimately enhancing patient care and long-term outcomes.

## CRedit authorship contribution statement

**Dr. Faten LIMAIEM and Dr Mouadh NEFISS:** Prepared, organized, wrote, and edited all aspects of the manuscript.

**Dr. Mouadh NEFISS,** and **Pr. Ramzi BOUZIDI:** Read, edited, and approved the final version of the manuscript. Contributed to data acquisition, analysis, and interpretation. Provided final approval of the manuscript before its submission.

## Consent

Written informed consent was obtained from the patient for publication of this case report and accompanying images. A copy of the written consent is available for review by the Editor-in-Chief of this journal on request.

## Ethical approval

Ethical approval for this study was provided by the Ethical Committee of the hospital.

## Guarantor

Dr. Faten LIMAIEM

## Provenance and peer review

Not commissioned, externally peer-reviewed.

## Sources of funding

This research did not receive any specific grant from funding agencies in the public, commercial, or not-for-profit sectors.

## Declaration of competing interest

None declared.
